# Primary Breast Angiosarcoma: A Case Report

**DOI:** 10.7759/cureus.91404

**Published:** 2025-09-01

**Authors:** Despoina Milonaki, Natalia Sinou, Nikoleta Sinou, Dimitrios Filippou, Ioannis Provatas

**Affiliations:** 1 Department of General Surgery, General Hospital of Nikaia "Agios Panteleimon", Athens, GRC; 2 Research and Education Institute in Biomedical Sciences, National and Kapodistrian University of Athens School of Medicine, Athens, GRC; 3 Department of Anatomy, National and Kapodistrian University of Athens School of Medicine, Athens, GRC; 4 Department of Pathology, General Hospital of Athens "Evangelismos", Athens, GRC

**Keywords:** angiosarcoma, breast, pathology, primary, radiation-associated

## Abstract

Primary breast angiosarcomas are extremely rare and aggressive tumors that originate from the inner lining of blood and lymphatic vessels in the breast. They are typically associated with a poor prognosis and have a strong tendency to metastasize. This report describes the case of a 57-year-old woman who detected a suspicious mass in her left breast during a routine self-examination. She initially underwent a lumpectomy, followed by a prophylactic mastectomy one year later. The tumor was identified as a well-differentiated primary breast angiosarcoma. The patient is currently being closely monitored and may require further treatment, including reconstructive surgery and adjuvant therapy. This case highlights the critical role of early detection and prompt intervention in enhancing the outcomes for patients with primary breast angiosarcomas. Despite their rarity, these tumors demand timely and comprehensive care to minimize the risk of recurrence and metastatic disease.

## Introduction

Angiosarcomas are uncommon tumors of mesenchymal origin that arise from the inner surface of blood and lymphatic vessels, exhibiting significant metastatic and aggressive behavior (also known as hemangiosarcoma and lymphangiosarcoma) [1]. These tumors can develop in any organ, but they are predominantly found in the skin and soft tissues. Notably, primary angiosarcoma of the breast is a malignant endothelial tumor originating in mammary tissue, typically not associated with prior radiation exposure. These cases are exceedingly rare, accounting for less than 1% of all breast cancer diagnoses [2]. Angiosarcomas can be categorized into primary and secondary types, with secondary angiosarcomas being more commonly encountered. Secondary angiosarcomas develop as a complication of radiation therapy after breast conservation or from chronic lymphedema. Primary angiosarcomas of the breast account for just 0.04% of malignant breast tumors [3]. The exact pathophysiological mechanisms behind primary angiosarcomas remain largely unclear [4].

Nevertheless, mutations in phospholipase C gamma 1 and kinase insert domain receptor, which are part of the vascular endothelial growth factor receptor 2 signaling pathway, have been noted. Additionally, a few exceptionally rare instances suggest amplification of the myelocytomatosis oncogene (MYC) gene. In contrast, secondary angiosarcomas in the breast are generally linked to prior radiation exposure in the chest region or adjacent areas.

This case report underscores the importance of maintaining a high index of suspicion and employing a meticulous diagnostic algorithm for these rare and aggressive masses. The difficulty in their diagnosis may lead to delayed or false treatment plans. This was the case of our patient, who required a two-stage surgical approach to prevent recurrence. Therefore, this report aims to raise concerns about best practices for diagnosis, treatment, and follow-up, while also highlighting the potential for conclusive preoperative diagnosis to prevent repeat surgical procedures. Sharing this case may contribute to the collective understanding and improve outcomes for patients with this challenging disease.

## Case presentation

A 57-year-old woman presented with a medical background of hypothyroidism and a previous cesarean section. She reports no allergies, does not smoke, and does not consume alcohol. She has been vaccinated against COVID-19 with three doses and has experienced two infections (Table 1).

**Table 1:** CARE checklist CARE: case report, COVID-19: coronavirus disease 2019, MRI: magnetic resonance imaging, BIRADS: Breast Imaging Reporting and Data System, PATTEY 1: Patey’s procedure type I, CD31: cluster of differentiation 31, CD34: cluster of differentiation 34

Patient information	57-year-old woman, history of hypothyroidism, cesarean section, no allergies, non-smoker, no alcohol use, COVID-19 vaccination (three doses), and two prior infections
Clinical findings	Palpable mass in the left breast discovered during routine check-up
Timeline	18/10/2023: biopsy → atrophy and stromal fibrosis, no malignancy. 30/10/2023: MRI → 7 × 6 × 5.2 cm mass, BIRADS 4. 24/11/2023: hospital admission. 27/11/2023: lumpectomy performed; biopsy → well-differentiated primary breast angiosarcoma. 29/02/2024: readmission for planned prophylactic mastectomy. 01/03/2024: radical mastectomy (left breast, PATTEY 1). 03/03/2024: discharge with REDOVAC and pain management plan
Diagnostic assessment	MRI of the breast. Histopathology: well-differentiated primary breast angiosarcoma. Immunohistochemistry: CD31/CD34 positivity
Therapeutic intervention	Lumpectomy, radical mastectomy (left breast), pain management post-surgery, future reconstructive surgery considered
Follow-up and outcomes	Patient under close surveillance, awaiting further biopsy analysis, potential adjuvant therapy may be required
Patient perspective	Patient detected breast mass during routine self-examination, sought medical evaluation promptly

**Figure 1:**
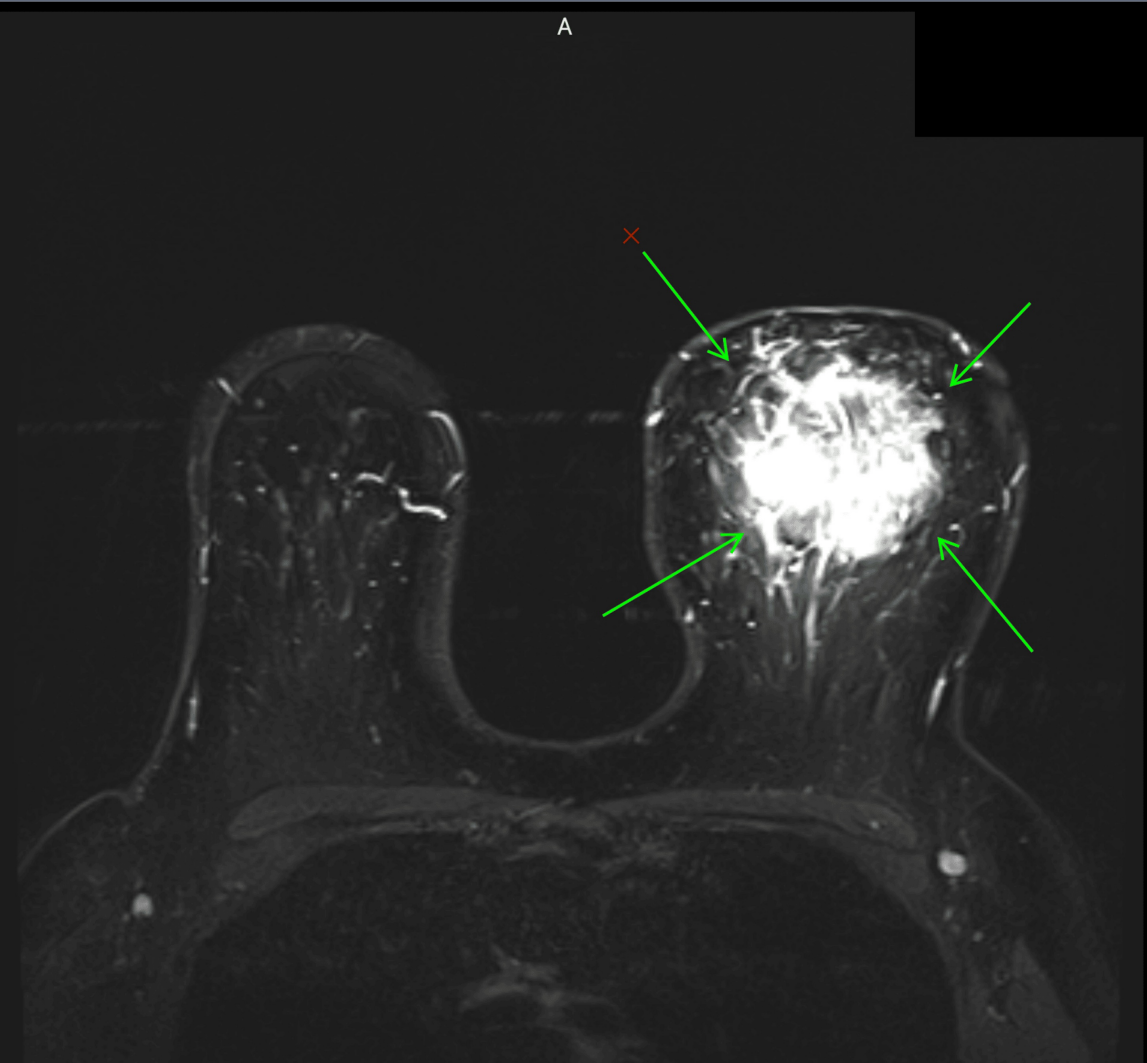
MRI MRI: magnetic resonance imaging

The patient was scheduled for admission to the hospital in November 2023 for surgical treatment of a left breast mass, which was identified one month prior during a routine check-up. An MRI conducted in October 2023 (Figure 1) revealed a sizable mass measuring 7 x 6 x 5.2 cm in the upper central area of the left breast, exhibiting atypical histological features categorized as Breast Imaging Reporting and Data System (BIRADS 4), warranting additional histological evaluation. Histological analysis of breast gland tissue samples taken in October 2023 revealed primary atrophy of the duct-lobular units, accompanied by moderate stromal fibrosis and a significant chronic inflammatory response. No signs of malignancy were found in the analyzed specimens.

The patient underwent a lumpectomy on the left breast at the upper outer quadrant (12 o’clock position). The procedure was conducted with the patient in the supine position and under general anesthesia. During this procedure, the mass identified measured approximately 7 × 6 × 5.2 cm. The lesion appeared infiltrative within the parenchymal tissue but without macroscopic involvement of the skin or nipple-areolar complex. The specimen was excised with surrounding breast tissue and sent for histopathological evaluation. On gross examination, the lumpectomy specimen revealed a multinodular, hemorrhagic mass with indistinct margins. The cut surface showed spongy, hemorrhagic areas interspersed with firm, grayish-white tissue, while the adjacent breast parenchyma demonstrated stromal fibrosis and chronic inflammatory changes. There were no complications, and the patient’s postoperative course was uneventful, with no concerning findings. The patient was discharged in a mobilized condition in November 2023 with detailed instructions and a pain management plan. A biopsy performed in November 2023 indicated a well-differentiated primary breast angiosarcoma. Due to this biopsy finding, the patient was admitted to the clinic in February 2024 for a planned prophylactic mastectomy of the left breast. In March 2024, the patient had a radical left breast mastectomy via PATEY 1 (modified radical mastectomy). The surgery was done with the patient in the supine position, the left arm in abduction and external rotation, under general anesthesia.

The entire breast was removed en bloc, including the previously affected region. Intraoperatively, no gross evidence of axillary lymph node involvement or direct skin infiltration was observed. The surgical specimen was sent for further biopsy analysis. The mastectomy specimen on gross examination contained a large hemorrhagic mass with ill-defined borders. The cut surface displayed heterogeneous features, with spongy and hemorrhagic regions alternating with denser gray-white areas and foci of necrosis. The surrounding breast tissue showed signs of fibrosis and chronic inflammatory reaction. The patient experienced no complications, and her postoperative course was uneventful. The patient was released in March 2024 with REDOVAC and a pain management plan in place, pending the biopsy results. Future reconstructive surgery may be necessary for the patient.

Histopathological findings

**Figure 2:**
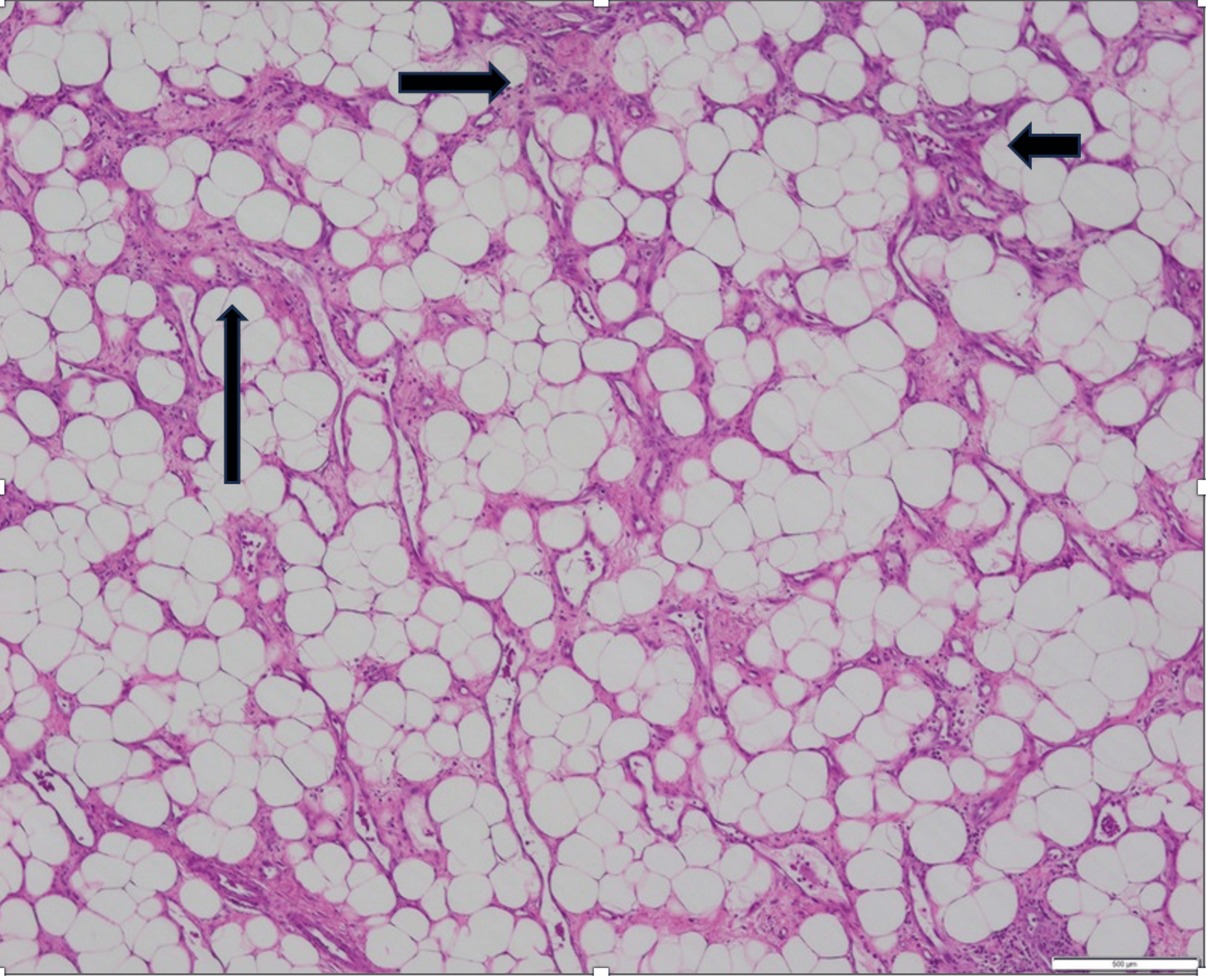
Primary breast angiosarcoma: well-structured small to medium anastomosing vessels, dissecting through adipose tissue (H&E stain 4x) H&E: hematoxylin and eosin

**Figure 3:**
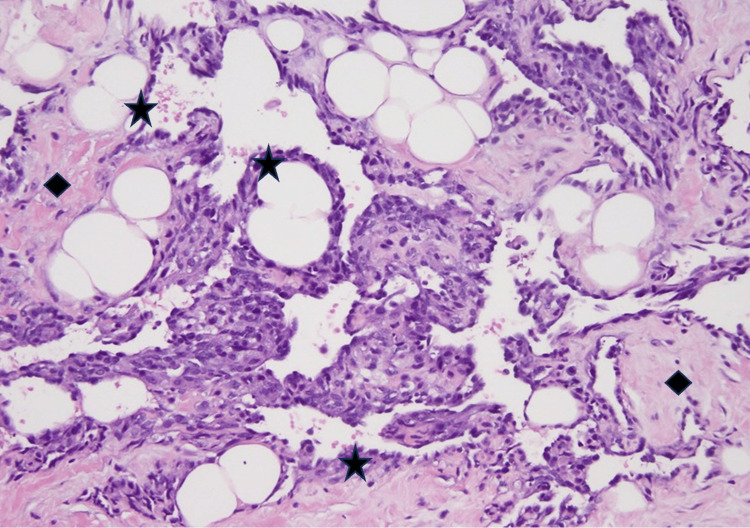
Primary breast angiosarcoma: irregularly vessels lined by flattened endothelial cells showing little atypical features (star) with moderate stromal fibrosis (square) (H&E stain 10x) H&E: hematoxylin and eosin

**Figure 4:**
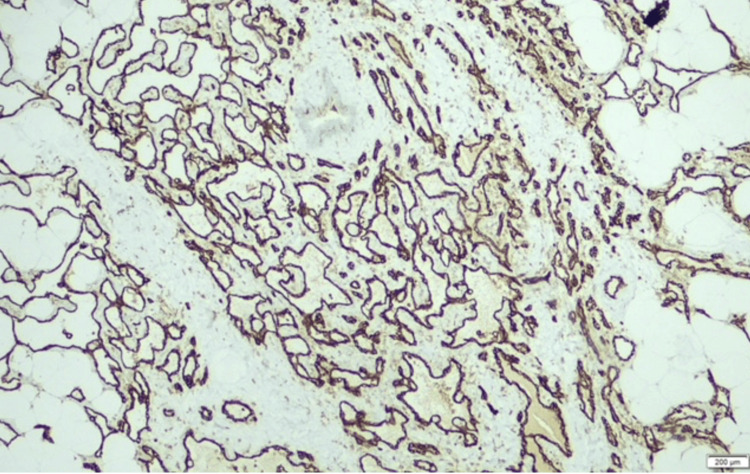
Primary breast angiosarcoma: CD34 diffuse positivity

**Figure 5:**
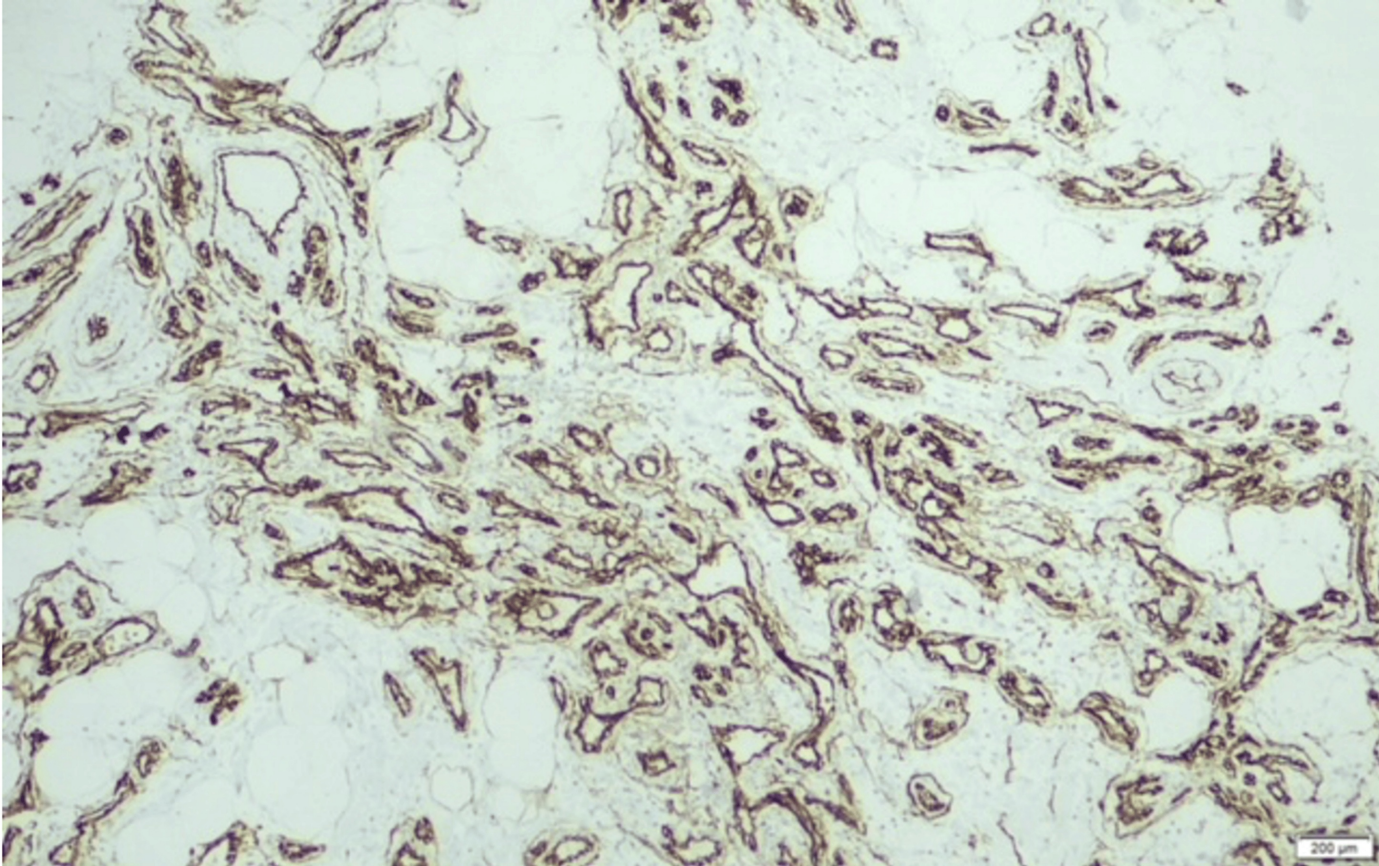
Primary breast angiosarcoma: CD31 diffuse strong positivity

Histological examination of the lumpectomy specimen disclosed a well-differentiated primary breast angiosarcoma. The tumor was constituted by irregularly shaped vascular channels dissecting through adipose tissue and mammary lobules, lined by flattened endothelial cells with minimal atypia. Areas of moderate stromal fibrosis and chronic inflammatory infiltration were also observed. The subsequent mastectomy specimen confirmed these findings, indicating well-structured small to medium anastomosing vessels infiltrating the fibroadipose tissue. Immunohistochemical staining demonstrated diffuse positivity for endothelial markers, including CD31 and CD34, as well as strong nuclear expression of ERG. These findings were consistent with the diagnosis of a well-differentiated primary breast angiosarcoma (Figures 2-5).

Follow-up

The patient has been under close surveillance following surgery. She underwent mammography, multiple CT scans (pan-CT), and PET-CT, all of which showed no evidence of recurrence or metastatic disease. Serial measurements of tumor markers have consistently remained within normal limits. Additionally, she undergoes regular hematological testing, which has also been unremarkable. To date, the patient’s follow-up course has been uneventful, with no radiological or laboratory findings suggestive of disease relapse. The patient has not yet undergone cosmetic reconstructive surgery.

## Discussion

Patients diagnosed with breast angiosarcomas typically have a median age of 40 years, in contrast to the 70-year median age seen in secondary angiosarcoma [3]. The patient's age of 57 is older than the median age of 40 typically seen in patients with primary angiosarcomas. Most individuals exhibit a rapidly enlarging mass, which is often accompanied by purplish-blue skin discoloration or breast asymmetry [5]. On mammography, it generally appears as a non-calcified mass or asymmetry [1]. Ultrasonography may show either a hyperechoic mass or a combination of hyper- and hypoechogenic areas accompanied by architectural distortion. Dynamic contrast-enhanced MRI displays characteristic patterns of malignant enhancement. Fine-needle aspiration has a false negative rate of 40%, which supports core needle biopsy as the preferred diagnostic method. Approximately 20% of patients are diagnosed with regional disease. The most common metastatic sites include the lungs, liver, bones, and central nervous system.

The tumors present as hemorrhagic, diffuse, or multinodular formations, ranging in size from 0.7 to 25 cm (average: 6.7 cm, median: 5 cm). Typically, they are found in parenchymal tissue rather than on the skin [6]. Their margins can often be indistinct. Tumors that are better differentiated exhibit a spongy, hemorrhagic aspect, while poorly differentiated tumors appear more solid, characterized by dense grayish-white tissue interspersed with necrotic areas. These less differentiated tumors may also contain regions of cystic, spongy, hemorrhagic vascular tissue in their vicinity.

Angiosarcomas are typically located deep within the breast tissue and may or may not extend to the skin, often exhibiting infiltrative or indistinct borders. The patient's mass was "sizable," measuring 7 × 6 × 5.2 cm. This size falls within the reported range for these tumors, which may range from 0.7 to 25 cm, with an average of 6.7 cm. The tumor was located in the "upper central area of the left breast," and it's reported that angiosarcomas are generally found deep within breast tissue.

A wide morphological spectrum is encountered, generally with a predominant vasoformative or solid component identified. Unlike secondary angiosarcomas, primary variants more frequently display well-defined, small- to medium-sized anastomosing vessels that penetrate fibroadipose tissue. Well-differentiated angiosarcomas consist of well-structured vessels lined by flattened endothelial cells with minimal atypia [7]. The presence of irregularly shaped vascular channels dissecting through adipose tissue and mammary lobules suggests a malignant nature. A lobular-type growth pattern, typically seen in hemangioma, is not present. Lesions with an intermediate appearance show endothelial multilayering, hobnailing, or papillary-like projections. Poorly differentiated angiosarcomas exhibit solid, cellular regions consisting of sheets of spindled to epithelioid cells interspersed with variably developed anastomosing vascular channels, accompanied by notable blood lakes, mitotic activity, and areas of necrosis [8]. Epithelioid angiosarcomas present a solid structure, characterized by sheets of large atypical epithelioid to polygonal cells that contain ovoid vesicular nuclei, prominent nucleoli, and relatively ample cytoplasm. Due to potentially limited vasoformation, epithelioid angiosarcoma can be mistaken for carcinoma. Frequent mitotic figures and necrosis are typically observed. Angiosarcomas express endothelial markers, with strong, membranous CD31 staining and nuclear ERG (ETS-related gene) immunoexpression noted. Aberrant expression of KIT, synaptophysin, chromogranin, and CD30 can be seen. Most tumors lack expression of the MYC protein. Staging currently has limited prognostic relevance.

The primary treatment for angiosarcoma is total mastectomy, with or without radiation therapy [9,10]. Nevertheless, local recurrences are noted in about 50% of patients. The choice of local treatment largely depends on the ratio of tumor size to breast size [11]. If the tumor is small and well-defined, it may be possible to excise it surgically with clear margins and opt for cosmetic reconstruction, making breast-conserving surgery a viable option. This is also the case for this specific patient, who may undergo cosmetic breast reconstruction at a later time. For patients with larger tumors, mastectomy is necessary. Axillary lymph node dissection is typically not performed unless there is pathological evidence of nodal involvement, given the low rates of nodal metastasis. The role of radiation following resection is not clearly defined [4]. Various studies often combine primary breast angiosarcomas with secondary (radiation-associated) angiosarcomas, employing differing approaches to radiation therapy and systemic treatment in either the adjuvant or neoadjuvant setting [12]. The primary chemotherapy treatment for breast angiosarcomas includes regimens based on taxanes and anthracyclines. In patients with locally advanced or metastatic angiosarcoma, the rate of complete or partial response was 25% (compared to 21% for those with other types of sarcomas). Enhanced progression-free and overall survival rates were observed with the combination of doxorubicin and ifosfamide, as opposed to using anthracycline chemotherapy alone [11].

Importance of this case

The significance of this case lies in its emphasis on the importance of early detection and prompt intervention in improving patient outcomes. Moreover, the case highlights the diagnostic challenges, as the initial histological analysis of the breast gland tissue did not reveal any signs of malignancy. It was only through a subsequent biopsy that the diagnosis was confirmed, leading to the necessary surgical treatment. The patient's age also provides a specific data point that contributes to the understanding of this rare disease, as it falls outside the typical median age range but is younger than the median age for secondary angiosarcomas.

## Conclusions

Primary mammary angiosarcomas are exceptionally rare malignant tumors known for their poor prognosis and high likelihood of metastasis. Although they represent only a tiny fraction of breast cancer cases, they present substantial difficulties in both diagnosis and treatment. Total mastectomy remains the primary approach for treatment due to its aggressive nature and tendency for early spread. However, even with surgical intervention, there is still a notable risk of recurrence, and adjunct therapies like chemotherapy and radiation often yield limited success. This underscores the importance of diligent postoperative monitoring and the need for advancements in treatment methods. Management of this rare but aggressive tumor type requires meticulous treatment planning from the initial diagnosis.
